# A novel nomogram based on complement C3 to predict the overall survival of early-stage hepatocellular carcinoma patients with microvascular invasion-positive undergoing curative resection

**DOI:** 10.3389/fonc.2025.1559083

**Published:** 2025-02-20

**Authors:** Guoyi Xia, Zeyan Yu, Shaolong Lu, Xiaobo Wang, Yuanquan Zhao, Jie Chen

**Affiliations:** ^1^ Department of Hepatobiliary Surgery, Guangxi Medical University Cancer Hospital, Nanning, Guangxi, China; ^2^ Department of Hepatobiliary Surgery, The Central Hospital of Shaoyang, Shaoyang, Hunan, China; ^3^ Department of Hepatobiliary Surgery, Guangxi Zhuang Autonomous Region People’s Hospital, Nanning, Guangxi, China

**Keywords:** hepatocellular carcinoma, early-stage, microvascular invasion, curative resection, overall survival

## Abstract

**Purpose:**

This investigation aimed to create a new nomogram based on complement C3 to forecast 1-, 3-, and 5-year overall survival (OS) rates in patients with early-stage hepatocellular carcinoma (HCC) exhibiting microvascular invasion (MVI) post-curative surgery.

**Methods:**

This study encompassed 1234 patients treated with resection at the Affiliated Cancer Hospital of Guangxi Medical University. The cohort for primary included 865 patients from December 2015 to December 2019, while the validation cohort comprised 369 patients. Follow-ups were conducted regularly until December 2024. Variables predicting survival were identified using Cox regression analyses, and based on these, a nomogram was constructed. This nomogram’s accuracy was assessed via time-dependent ROC curves, calibration curves and KM curve analyses.

**Results:**

Investigations identified complement C3, PT, the presence of cirrhosis, tumor capsule, and MVI-M2 as distinct predictors of survival in HCC patients. Based on these findings, a predictive nomogram was constructed and validated, aimed at estimating the 1-, 3-, and 5-year OS. The efficacy of the nomogram was validated through analyses with ROC curves, calibration curves, each demonstrating positive outcomes. Additionally, KM curve analysis effectively separated the patient populations into two prognostic risk categories within both the primary and validation cohorts.

**Conclusion:**

In conclusion, a new nomogram has been developed and corroborated through multivariate Cox regression analysis, aimed at estimating overall survival for patients in early stages of microvascular invasion following surgical resection. This tool has proven to be more effective in forecasting survival outcomes for such patients post-curative surgery.

## Introduction

Hepatocellular carcinoma (HCC), recognized as the most prevalent type of primary liver malignancy, has been observing a global increase in incidence (1). Data from 2022 report approximately 865,269 new instances and 757,948 fatalities due to HCC, underscoring the severe impact of this disease. The major risk factors contributing to the disease include infection by hepatitis B and C viruses, chronic alcohol abuse, the development of nonalcoholic fatty liver disease (NAFLD), and the carcinogenic effects of aflatoxins (2). Notably, China is responsible for nearly half of the reported cases and deaths, reflecting a generally grave prognosis for the disease across various populations (3). For patients diagnosed in the early stages, the curative interventions primarily include surgical resection, complemented by options like local ablative therapies and liver transplantation, with resection being the cornerstone of treatment (4, 5). Despite these available treatments, the heterogeneity among HCC tumors presents significant challenges, resulting in considerable variability in overall survival rates among patients who undergo surgery. In practice, these treatments prove effective in only 15%-30% of cases, and the five-year survival rate after diagnosis remains alarmingly low at just 18% (6, 7).

Microvascular invasion (MVI), identified as the presence of tumor cells within the tiny blood vessels of the liver tissue surrounding a tumor, is an influential prognostic factor in HCC. This condition is subdivided into two classifications: M1, where up to five microvascular invasions are found within 1cm of the tumor margin; and M2, characterized by more than five microvascular invasions or invasions beyond 1cm of the tumor edge (8). MVI is widely recognized as a critical indicator of increased risk for tumor recurrence and a poor prognosis in HCC patients (9, 10). Notably, about 30% of patients diagnosed with early-stage HCC are identified as MVI-positive, which is strongly linked to the early recurrence of the disease (11). However, the current research on prognostic models for early-stage HCC that consider MVI is insufficient, limiting a comprehensive understanding of the long-term survival and recurrence risks associated with these patients (12, 13). Consequently, there is an urgent need for extensive, long-term studies that involve larger patient cohorts and gather more expansive data to deepen our comprehension of long-term outcomes for HCC patients affected by MVI. Such research will enhance the decision-making tools available to healthcare professionals.

Recent serological assessments have identified complement C3 as a significant independent factor in forecasting the outcomes of radical surgeries in various cancers, including esophageal, gastric, and breast cancers (14, 15). Furthermore, nomograms incorporating complement-related genetic markers have demonstrated some capacity to predict outcomes effectively (16). However, research into the effectiveness of complement C3 as a serological marker for forecasting the prognosis of hepatocellular carcinoma remains notably scarce.

Presently, numerous investigations have leveraged nomograms to forecast overall survival (OS) in HCC patients following surgical interventions (17–19). Despite these advancements, research specifically targeting early-stage HCC patients who test positive for MVI remains sparse. It is therefore imperative to swiftly pinpoint critical risk factors and establish an all-encompassing predictive model for OS within this distinct subgroup. Such a model would facilitate the timely provision of suitable treatment strategies, significantly enhancing patient outcomes.

## Patients and methods

### Patients

**Figure 1:**
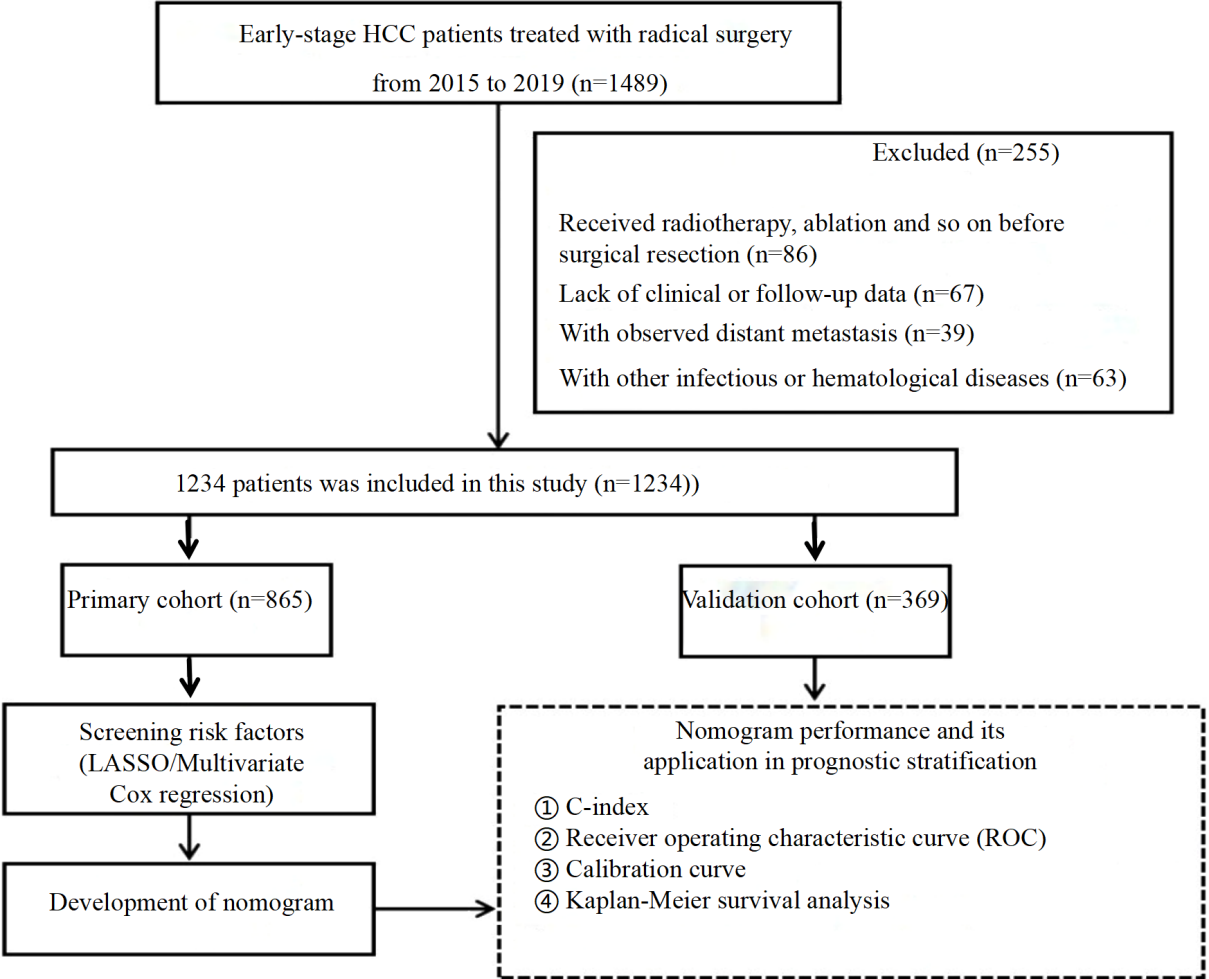
Flow chart of patients screening and grouping.

From December 2015 to December 2019, a comprehensive dataset was compiled from 1,234 patients who underwent curative resections for early-stage HCC at the Affiliated Cancer Hospital of Guangxi Medical University. Inclusion criteria required individuals to have a confirmed diagnosis of primary HCC via pathological examination, classified at the BCLC stage 0/A, and treated initially with surgical resection. Additionally, pathology reports must indicate an MVI classification of M1 or M2, and there should be extensive clinical and follow-up data available for each case. The study excluded patients with secondary tumors, those who had previously undergone radiotherapy, chemotherapy, or ablation treatments, those lacking adequate clinical or follow-up documentation, those diagnosed with distant metastasis indicating a progression beyond early-stage disease, and those suffering from concurrent infectious or hematological conditions. After meeting the specific and strict inclusion criteria, the patients were randomly assigned to one of two groups: the primary cohort or the validation cohort. A computerized randomization system was used to randomly allocate participants in an appropriate distribution. The primary cohort was allocated at 70% and 30% for the validation cohort. In total, this provided 865 patients in the primary cohort and 369 in the validation cohort. **Figure 1**, a flowchart of the entire process of patient recruitment and the organizational structure of the study, illustrates such organizational intricacies. Due to the retrospective study nature, the study was approved by the Ethics Committee of the Affiliated Cancer Hospital of Guangxi Medical University. The Declaration of Helsinki ethical standards and guidelines were followed. With regard to the design of such retrospective studies, and against the background of the Helsinki Declaration, the local ethics committee declared the requirement of written informed consent by the patients unnecessary. Retrospective research protocols hold the established exemption of the requirement of direct patient consent, and this exemption fits within this framework.

### Data collection

For the purposes of this research, a comprehensive baseline characteristics compilation for patients ready to undergo curative surgical resections for early stage hepatocellular carcinoma was undertaken. This preparatory phase included the acquisition of key personal and medical data grouped into several distinct categories: (1) Personal demographics included each patient’s age and gender; (2) Diagnostic imaging and pathological evaluations informed each case, including findings which showed cirrhosis, ascites, and portal hypertension. Additionally, tumor characteristics were assessed including size and number of tumors; and (3) a variety of blood tests were accomplished to measure a comprehensive biochemical and immunological profile of each patient. These included measurements of D-dimer, complement C3 and measures of hepatitis. In immune cell analysis, helper T cells (Th), suppressor T cells (Ts), natural killercells (NK), B-lymphocytes (B-Lym), general lymphocytes (Lym), neutrophils (Neu), monocytes (Mon), and platelets (PLT), were all covered. Moreover, alanine aminotransferase (ALT), aspartate aminotransferase (AST), total bilirubin (TBIL), total cholesterol (TC), prothrombin time (PT), carcinoembryonic antigen (CEA), and alpha fetoprotein (AFP) were analyzed as key liver function tests; (4) Pathological examinations was also done to observe the tumor capsule, the Edmondson stage of the tumor, as well as to assess the grade of microvascular invasion.

### Follow-up

OS is an important clinical endpoint metric which measures the time to an event after a specific starting point, namely date of curative surgery for hepatocellular carcinoma in this instance, to death of either the patient or date of the most recent follow up contact. Patients in this study were monitored for HCC post surgically, commencing one month after treatment, followed by quarterly monitoring for one year. Evaluations were next scheduled every six months until death or final follow up. These assessments, routinely performed using radiological imaging techniques, such as CT and MRI, provide regular monitoring of tumor size and detection of any arising irregularity. Furthermore, recurring blood tests are done to test the liver functionality as well as tumor marker, to identify abnormalities in the biochemical markers. Follow up of the study ended on December 30, 2024 (with a median of 36.0 months of follow up, calculated by the reverse Kaplan Meier method).

### Statistical analysis

All statistical analyses in this research were performed using R software version 4.3.1, incorporating various essential packages such as “rms”, “glamet”,”rmda”, “survival”, “survminer”, and “ggplot2”. To validate the model comprehensively, subjects were allocated randomly into a primary cohort and a validation cohort, with a proportion of 70% to 30%, respectively. The primary cohort was used for model development, and the validation cohort tested its efficacy. Categorical variables were analyzed with the Chi-Square or Fisher’s exact test, and continuous variables with the t-test or Mann-Whitney U test. The Lasso and multivariate Cox regression identified key prognostic indicators. Significant factors were used to construct nomograms for predicting 1-year, 3-year, and 5-year OS, evaluated by C-index, ROC curves, calibration plots. Survival outcomes were compared using Kaplan-Meier curves and log-rank tests, stratifying patients into risk categories.

## Results

### Baseline characteristics

In this investigation, a comprehensive enrollment of 1234 early-stage HCC patients who underwent curative resection was achieved. These participants were systematically segregated into two distinct groups: the primary cohort comprised of 865 patients, and the validation cohort, which included 369 patients. The baseline characteristics of patients across both groups are detailed in (**Table 1**), which indicates uniformity across the cohorts as evidenced by the lack of statistically significant disparities in baseline variables (p > 0.05).

**Table 1:** Patient clinical characteristics in the primary cohort and validation cohort.

Characteristics	Primary cohort *N=865*	Validation cohort *N=369*	p.overall
Age(mean±SD),year	51.7 (11.1)	52.8 (11.6)	0.109
D.dimer(mean±SD),µmol/L	0.86 (1.53)	0.88 (1.35)	0.838
C3(mean±SD),g/L	0.92 (0.24)	0.94 (0.26)	0.128
Th(mean±SD),%	39.5 (7.71)	39.4 (7.83)	0.878
Ts(mean±SD),%	21.1 (6.44)	21.0 (6.96)	0.887
NK(mean±SD),%	14.2 (22.3)	13.8 (7.64)	0.606
B.Lym(mean±SD),%	13.6 (5.72)	13.5 (5.71)	0.929
TBIL(mean±SD),µmol/L	15.5 (13.1)	16.6 (24.3)	0.414
ALB(mean±SD),g/L	38.7 (4.31)	38.4 (4.25)	0.141
ALT(mean±SD),u/L	42.4 (41.1)	39.0 (33.0)	0.132
AST(mean±SD),u/L	45.8 (29.5)	46.2 (31.9)	0.867
TC(mean±SD),mmol/L	4.85 (1.17)	4.88 (1.19)	0.661
Platelet(mean±SD),10^9^/L	198 (74.7)	198 (80.5)	0.922
Neutrophil(mean±SD),10^9^/L	3.66 (1.50)	3.71 (1.59)	0.647
Monocyte(mean±SD),10^9^/L	0.48 (0.32)	0.52 (0.50)	0.221
Lym(mean±SD),10^9^/L	1.83 (1.26)	1.95 (2.14)	0.320
CEA(mean±SD),ng/mL	2.89 (5.18)	5.08 (37.5)	0.265
PT(mean±SD),S	12.6 (1.27)	12.5 (1.35)	0.341
AFP(%)			0.562
>400 ng/mL	293 (33.9%)	118 (32.0%)	
≤400 ng/mL	572 (66.1%)	251 (68.0%)	
Hepatisis(%)			0.037
HBV-negtive	397 (45.9%)	194 (52.6%)	
HBV-positive	468 (54.1%)	175 (47.4%)	
Gender(%)			0.920
Female	130 (15.0%)	57 (15.4%)	
Male	735 (85.0%)	312 (84.6%)	
Portal.H(%)			0.260
No	756 (87.4%)	313 (84.8%)	
Yes	109 (12.6%)	56 (15.2%)	
Ascites(%)			0.626
>20ml	64 (7.40%)	31 (8.40%)	
≤20ml	801 (92.6%)	338 (91.6%)	
Tumor.size(%)			0.275
>5cm	460 (53.2%)	183 (49.6%)	
≤5cm	405 (46.8%)	186 (50.4%)	
Cirrhosis(%)			0.886
No	255 (29.5%)	111 (30.1%)	
Yes	610 (70.5%)	258 (69.9%)	
Tumor-number(%)			0.902
Multiple	104 (12.0%)	46 (12.5%)	
Single	761 (88.0%)	323 (87.5%)	
Tumor-capsule(%)			0.517
Complete	636 (73.5%)	264 (71.5%)	
Incomplete	229 (26.5%)	105 (28.5%)	
Edmondson-stage(%)			0.290
I-II	512 (59.2%)	231 (62.6%)	
III-IV	353 (40.8%)	138 (37.4%)	
MVI(%)			0.810
M1	545 (63.0%)	244 (66.1%)	
M2	320 (37.0%)	125 (33.9%)	

AFP,alphafetoprotein;C3,complement C3;Th,helper T cells;Ts,suppressor T cells;NK, natural killercells; B-Lym,B-lymphocytes;Lym,general lymphocytes;Neu, neutrophil; Lym, lymphocytes; Mon, monocyte; PLT, platelet; ALT, alanine aminotransferase;AST,aspartate aminotransferase; TBIL: total bilirubin;TC,total cholesterol;PT,prothrombin time;CEA,carcinoembryonic antigen;MVI,microvascular invasion.

### Screened risk factors for overall survival

**Figure 2:**
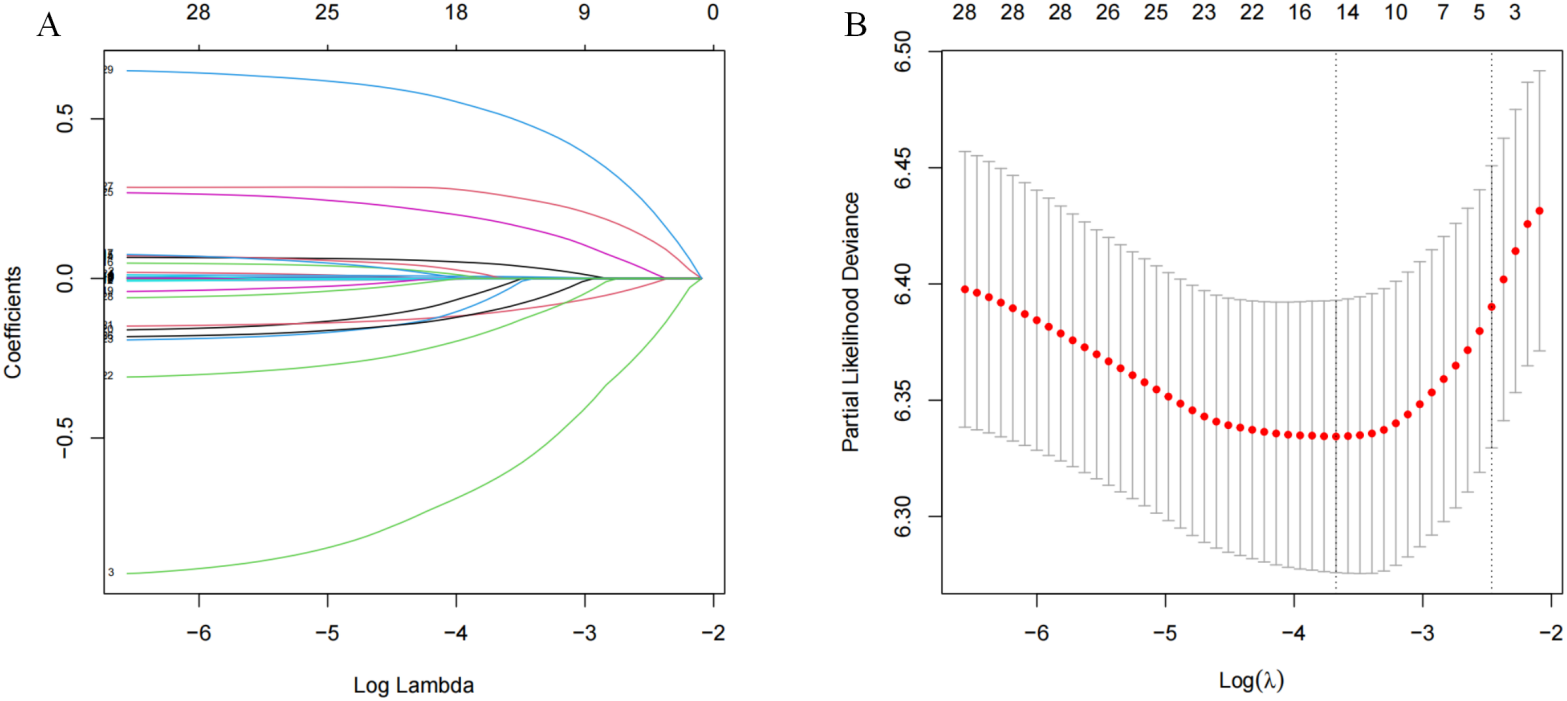
Lasso regression analysis in the primary cohort. **(A)** Variation features of the coefficient of variables; **(B)** Determination of the optimal value of λ through cross-validation method.

The LASSO coefficient profiles of the features were plotted. The optimum parameter (lambda) selection in the LASSO model performed tenfold cross-validation through minimum criteria. The partial likelihood deviance (binomial deviance) curve is presented versus log (lambda) (**Figure 2A**). Dotted vertical lines are shown at the optimum values by performing lambda.min and lambda.1se (**Figure 2B**). Finally, gender,ascites.portal hypertension, tumor-number, cirrhosis, C3,CEA,AST,PT,Th,B.Lym, monocyte, neutrophil, tumor-capsule and MVI were selected according to the optimum value corresponding to the minimum value of lambda (**Figure 2**). Then, we used the multivariate Cox regression to further identify the most crucial variables essential for OS prediction, and the results were displayed (**Table 2**). Cox regression is based on a semiparametric model, which assumes that the effect of predictor variables on time of event (death) is described by a hazard proportional function. Therefore, the results of Cox regression are usually presented in the form of hazard ratio (HR). In our analysis, it was revealed that cirrhosis (HR: 1.26; 95% CI: 1.05 - 1.5; P=0.011), C3 (HR: 0.49; 95% CI: 0.34 - 0.71; P<0.001),PT(HR: 0.86; 95% CI: 0.81-0.92; P<0.001), tumor-capsule(HR: 1.39; 95% CI: 1.16 - 1.66; P<0.001), MVI-M2(HR: 1.9; 95% CI: 1.56-2.32; P<0.001) were the most significant variables for OS prediction. Of the variables, cirrhosis, tumor-capsule, and MVI -M2 were identified as having a hazardous effect, whereas C3 and PT were considered to have a protective effect.

**Table 2:** Univariate and multivariate Cox analysis of the primary cohort.

Variables	Univariate analysis			Multivariate analysis		
	HR	95%CI	P.value	HR	95%CI	P.value
Gender	0.91	0.74-1.14	0.417			
Ascites	0.81	0.59-1.13	0.217			
Portal.H	0.78	0.61-1.01	**0.055**			
Tumor-number	0.8	0.64-1.01	**0.063**			
Cirrhosis	1.35	1.14-1.6	**0.001**	1.26	1.05-1.5	**0.011**
C3	0.46	0.32-0.67	**<0.001**	0.49	0.34-0.71	**<0.001**
CEA	1	0.99-1.01	0.907			
AST	1	1-1	**0.081**			
PT	0.9	0.84-0.96	**0.001**	0.86	0.81-0.92	**<0.001**
Th	1.01	1-1.02	0.103			
B.Lym	0.99	0.98-1.01	0.403			
Monocyte	1.09	0.91-1.3	0.374			
Neutrophil	1.05	1-1.11	**0.074**			
Tumor-capsule	1.51	1.27-1.81	**<0.001**	1.39	1.16-1.66	**<0.001**
MVI	1.84	1.51-2.24	**<0.001**	1.9	1.56-2.32	**<0.001**

Bold represents statistical significance, with univariate Cox <0.1 and multivariate Cox <0.05.

### Development of the nomogram for overall survival

**Figure 3:**
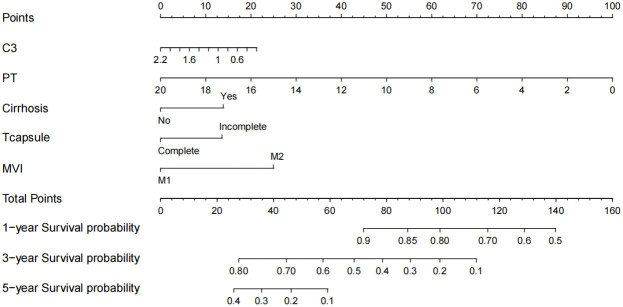
Nomogram for predicting the 1-, 3-, and 5-year overall survival (OS). C3, complement C3; PT, prothrombin time; MVI, microvascular invasion.

Using the five previously identified critical prognostic factors, a sophisticated nomogram capable of graphically representing 1 year, 3 year, and 5 year OS rates of patients with early stage hepatocellular carcinoma who undergone curative surgical intervention is constructed (**Figure 3**). Each prognostic factor was scored and then aggregated into a composite score, plotted on a comprehensive range of a total points scale. This scale has proved to demonstrate the projected survival probabilities at these intervals and provides a detailed prognostic for clinical outcomes.

### Performance of the established nomogram

**Figure 4:**
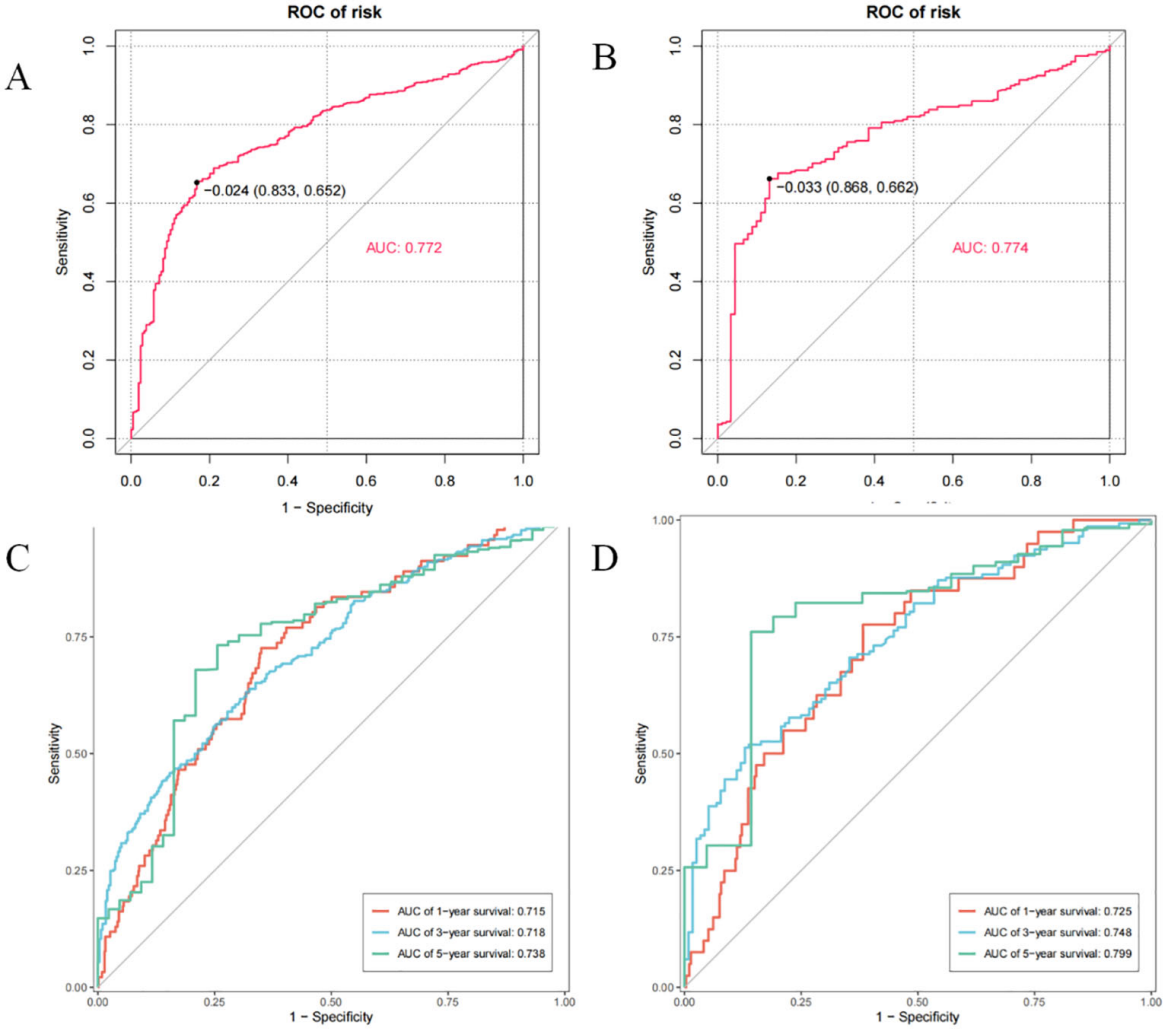
The C-index and time-point roc curves of the nomogram in the primary and validation cohort. **(A)** The C-index in the primary cohort. **(B)** The C-index in the validation cohort. **(C)** The AUCs for OS at 1, 3and 5 years in the primary cohort. **(D)** The AUCs for OS at 1, 3and 5 years in the validation cohort.

**Figure 5:**
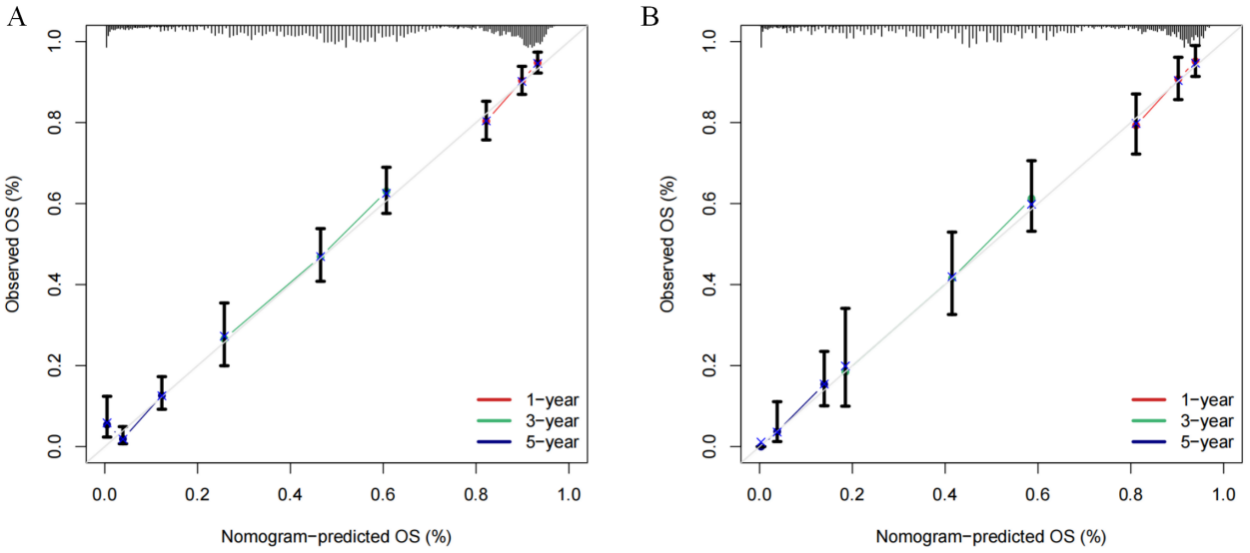
Calibration curves of the nomogram for predicting the 1-, 3-, and 5-year overall survival (OS) in the cohorts. **(A)** Calibration curves in the primary cohort. **(B)** Calibration curves in the validation cohort.

In primary cohort, the nomogram achieved a C-index of 0.772 with a 95% confidence interval spanning from 0.652 to 0.833 (**Figure 4A**). This result demonstrates the model’s proficiency in distinguishing between individuals with differing survival times. Subsequently, we developed time-dependent ROC curves for predicting 1-year, 3-year, and 5-year OS within the primary cohort (**Figure 4C**). The analysis revealed that the Area Under the Curve (AUC) values for the 1-year, 3-year, and 5-year OS predictions were 0.715, 0.718, and 0.738, respectively. These AUC values underscore the model’s strong predictive performance. The calibration curves for 1-year, 3-year, and 5-year OS probabilities demonstrated a high level of concordance between the observed clinical outcomes and the survival probabilities estimated by the nomogram (**Figure 5A**). This consistent accuracy in survival predictions at various time points enhances the nomogram’s utility in personalized patient care.

### Validation of the nomogram

The validation cohort achieved a C-index of 0.774 (**Figure 4B**), with a 95% confidence interval spanning from 0.662 to 0.868, which reflects a superior level of discriminative power. Additionally, the AUC values for OS at 1-year, 3-year, and 5-year intervals were significantly elevated, recording scores of 0.725, 0.748, and 0.799, respectively (**Figure 4D**). These elevated AUC values demonstrate the nomogram’s exceptional ability to differentiate between patients who are likely to survive beyond specific time points and those who are not. Such findings are crucial in establishing the nomogram’s high predictive accuracy. Furthermore, the calibration curves for 1-year, 3-year, and 5-year OS indicated that the nomogram’s predicted probabilities closely matched the actual observed outcomes (**Figure 5B**), thereby confirming its strong consistency and reliability.

### Prognostic stratification of the patients based on the nomogram scores

**Figure 6:**
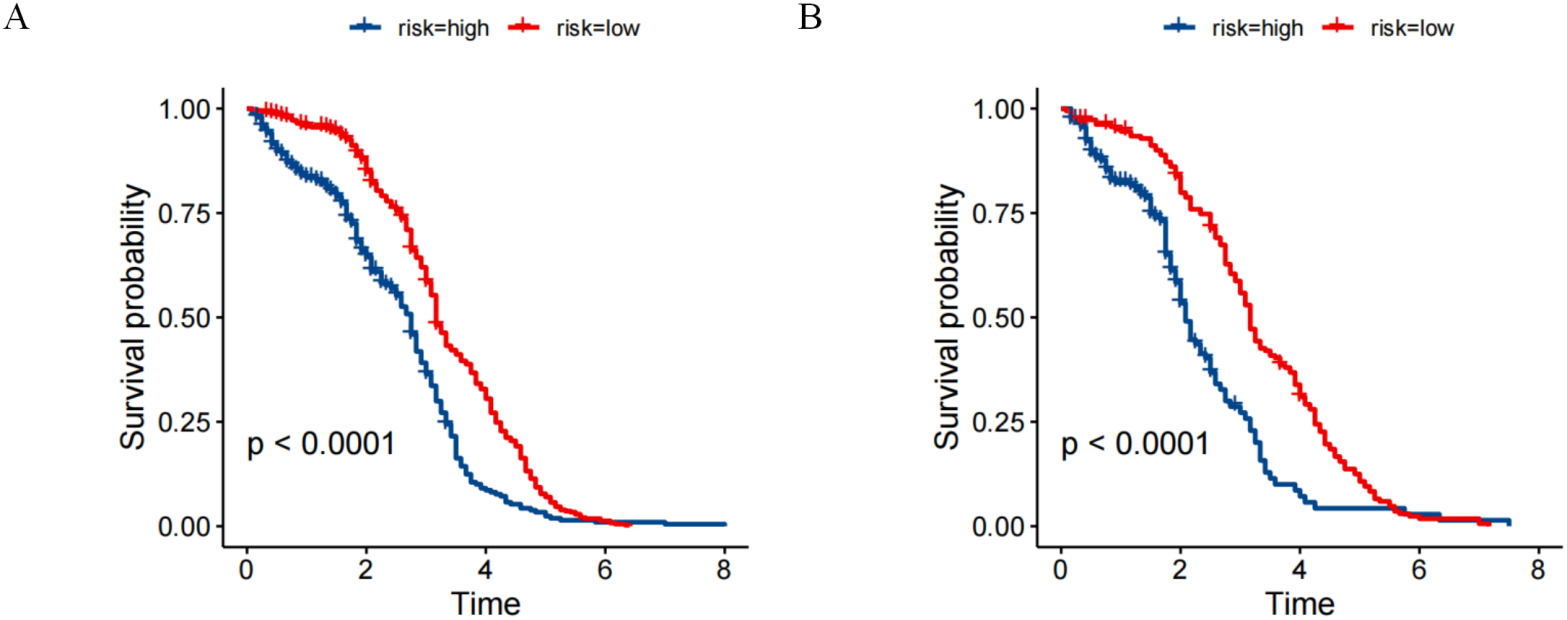
Risk stratification for overall survival (OS) based on the nomogram risk scores **(A)** primary cohort. **(B)** validation cohort.

Utilizing the aggregate scores generated by the nomogram, a detailed risk stratification framework was developed, effectively dividing patients into two principal categories: low-risk and high-risk. The implementation of Kaplan-Meier survival analysis illuminated the significant implications of this stratification approach. The analysis outcomes demonstrated a marked separation in survival probabilities between the two identified risk groups. Specifically, within the primary cohort, individuals categorized under the low-risk group exhibited substantially higher survival rates over the study period, whereas those assigned to the high-risk category faced considerably poorer prognoses (**Figure 6A**). Furthermore, the analysis conducted on the validation cohort replicated and reinforced the findings observed in the primary cohort (**Figure 6B**). All results showing statistical significance (P < 0.001) (**Figure 6**).

## Discussion

This work investigates an integrated Lasso regression with multivariate Cox proportional hazards modelling for a thorough assessment of the initial demographic and clinical profiles of the patient cohort. After a thorough evaluation, a prognostic nomogram was meticulously created and proven to reliably predict OS rates out to one, three, and five years following a surgical cure for early stage HCC patients with MVI who undergo surgical curative resection. Accurate prognostic assessment and the precision of survival prediction and appointment of effective post-treatment patient management strategies was improved using the developed nomogram.

Several distinct advantages of this investigation over previous studies examining prognostic outcome of early stage HCC patients after curative surgical resection are then identified. Secondarily, the study narrowed its patient population to MVI positive patients. HCC patients with MVI are recognized as an important prognostic factor with significant effects on early recurrence rates and longterm survival outcomes (20). Since MVI are present in about 30% to 50% of persons diagnosed with early stage HCC, these patients represent an important subgroup that should be examined in order to identify the prognostic determinants for their survival. Furthermore this research has an extended sample cohort as a second strength. To that end, we obtained comprehensive data for a large group of early-stage HCC patients who underwent curative resection, composed of clinical information, pathological characteristics, and survival metrics. Due to the large size of this cohort, this cohort allows for more reliable comparison (with greater degrees of freedom) between patient groups and therefore increases reliability and validity of predictive model (laboratory changes importance). Furthermore, the relatively large sample size reduces the possibility of model overfitting, guaranteeing higher generalizability of our predictive results. Rigorous protocols for maintaining the quality and accuracy of the data were realized through the data collection phase. A standardized data collection framework was developed in terms of timing of data collection, data sources, and manner of data presentation in order to achieve consistency and precision. Data verification for subsequent data included data consistency cheque, outlier identification and missing data management. Data cleaning procedures were performed when necessary to remove abnormal or unreasonable entries and/or to correct or complete incomplete data points when needed. In addition, data sampling and random audits were performed for quality assessments to notice potential problems so corrective actions could be done in a timely manner. Third, the study has long term follow up data that is essential in explaining the prolonged survival of patients after treatment. To capture accurately the long term effectiveness of curative resection on patient outcomes, our research team undertook years of follow up after resection. Lastly, this study provides an important advantage of use of machine learning techniques in conjunction with traditional statistical methods. What machine learning algorithms can do is they can handle a lot of data, and they can find complex relationships between one thing and another in that data. In particular, we conducted Lasso regression to discover prognostic factors and integrated these with multivariate Cox regression analyses to build a better survival prediction model. The hybrid approach promotes a more nuanced understanding of the complex associations between patient survival and numerous potential factors, and supports a strong basis for customized treatment strategies.

Researchers have introduced numerous staging frameworks to improve evaluation of survival outcomes for patients with HCC (21, 22). Despite this, the majority of these existing frameworks lack the ability to capture the nuances that go along with certain HCC treatment modalities, requiring more of a complex assessment system. As far as we are aware, the nomogram developed in this study is the first to concurrently incorporate five unique factors: We developed a predictive model (C3, PT, cirrhosis, tumor capsule, and MVI-M2) to predict OS in patients with early stage HCC after curative resection. Because these parameters are readily obtainable from standard clinical evaluations, there is no additional expense and little burden on the patient. Unlike previous nomograms, besides conventional imaging and pathological variables (i.e., cirrhosis and tumor size, number, resectability and location) our nomogram incorporates serological biomarkers including C3 and PT. As a result, this nomogram is an innovative approach in overcoming the shortcomings of current staging systems, to predict more whole and individualized prognosis to individuals with hepatocellular carcinoma.

For this investigation, we incorporated major nodal points from both the complement and coagulation pathways, complement C3and PT, into the nomogram model. The complement C3, a plasma protein, produced by the liver and macrophages is an important part of the complement cascade (23). The complement system is an important component of the immune system and can be activated through multiple pathways and plays a vital role in response to cellular damage independent of pathogens, but functions in a dual manner in liver injury exacerbating and mitigating it (24, 25).Activation of the complement cascade is implicated in many hepatic conditions such as alcohol related liver disease, metabolic steatosis, fibrosis, autoimmune liver disorders, and hepatocellular carcinoma, and uncontrolled activation of complement cascade play a role as pathogenic mediator in progression of liver diseases (26).Diverse antigen antibody complexes bring about complement system activation and excessive depletion of complement components, C3 for example, in HBV infection related hepatocellular carcinoma (27).Furthermore, the results show that PIWIL1 induces HCC cells to secrete complement C3, which promotes HCC development by interacting with myeloid derived suppressor cells (MDSCs), and promotes the production of the immunosuppressive cytokine interleukin 10 (IL-10). The immunosuppressive activity of PIWIL1 influenced MDSCs in the HCC tumor microenvironment is diminished by an inhibition of IL-10 secretion which leads to a promotion of hepatocellular carcinoma (HCC) initiation, development and progression (28).The imbalance of the complement system, with excessive activation and consumption, can change the microenvironment of the tumor of hepatocellular carcinoma cells causing development and progression of the tumor (29).The present study determined that the hazard ratio (HR) for complement C3 with values below one indicates a protective factor. One reason for this observation is the fact that the majority of the recruited population are infected with HBV leading to excessive consumption of complement components.

Cirrhotic patients often have an extended PT, due to decreased liver production of coagulation factors. On the other hand, we found that the HR for our PT is less than one, indicating it acts as a protective element. Yugawa K (30) also showed that decreased PT is correlated with a low ratio of lymphocytes to C reactive protein (CRP), which had been associated with poorer outcomes in HCC patients. An increased PT has been widely accepted as an indicator of advanced stage of cirrhosis, but the association between PT and HCC remains unrevealed and needs further study.

Cirrhosis and incomplete tumor capsule are reported as significant risk factors for the presence of MVI, and are associated with worse prognostic outcomes in HCC patients (31–33). Our results confirm these findings, and we find that patients with these features are more likely to develop more aggressive tumor forms and have a tumor microenvironment that is bad for patient prognosis.

M2 states that if there are more than five MVIs, or the MVIs are more than one centimeter from the primary tumor, the tumor is more adhesive, infiltrative and also able to breach tissues surrounding it (34).Previous studies have identified vascular endothelial growth factor A (VEGF-A) and stathmin 1 (STMN1) as biomarkers associated with different grades of MVI. Wang (35) discovered that patients classified as M2 exhibited the highest average serum concentrations of VEGF-A. Cai (36) demonstrated that STMN1 levels in HCC tissues rise progressively with higher MVI grades (M1, M2), and these findings reached statistical significance. Both VEGF-A and STMN1 are recognized as crucial biological markers that contribute to the advancement and poor prognosis of liver cancer. Additional research has established that the severity of MVI grading is independently correlated with OS following radical resection in early-stage HCC, particularly in patients classified as M2 (37).In our study, the HR for MVI-M2 was determined to be 1.9, with a 95% CI ranging from 1.56 to 2.32, and a P-value of less than 0.001. This indicates that MVI-M2 serves as an independent risk factor influencing overall survival, aligning with previous research findings. Furthermore, a propensity score analysis revealed that the combination of lenvatinib and PD-1 inhibitors administered post-surgery significantly enhanced the prognosis of HCC patients with early MVI, especially those categorized as MVI-M2, suggesting potential benefits from adjuvant therapy (38).

In conclusion, the integration of the five previously identified risk factors into nomograms significantly enhances the accuracy of predicting overall survival in early-stage HCC patients with positive MVI. The performance of the nomograms in evaluating and modelling therapeutic efficacy and treatment adverse effects in individual patients is shown, in addition to classifying patients into distinct risk categories, allowing high risk patients for whom such a classification is established to choose between equally efficacious treatment modalities to favor those with low adverse effects and the other group to prefer treatment modalities that offer low efficacy with low treatment adverse effects. This custom approach to treatment selection improves survival outcomes and also results in more effective interventions and fewer instances of unnecessary complications. In addition, this technique optimizes the usage of medical resources which attenuates the total load of the healthcare system.

Our study has many limitations. Firstly, the development and validation of nomogram relied only on data of a single center in China. And, this limitation might deconstrain the generalizability of our findings to broader populations. Accordingly, future studies should include external validation and prospective randomized controlled trials to confirm and validate our results. Secondly, our findings can only be applied to early stage HCC patients who underwent curative surgery. Further research is required to realize the relevance of nomogram to other patient groups or treatment modalities. The nomogram outing can be used to help clinical decision making by identifying those at highest risk and guiding the selection of appropriate therapeutic strategies. Yet this data must be explored more thoroughly, since there is not a current wide dataset of this. Such data could be integrated into future investigations to yield a more thorough interpretation of the factors that determine HCC outcomes, which may improve the quality and relevance of our findings in clinical practice. Thirdly, the majority of patients in this study had HBV infection associated HCC, a trend that is becoming less common due to the rising incidence of non HBC etiologies. This shift may change the behavior of tumor markers and thus the generalizability of our results to other types of HCC patients, mostly HBV-associated HCC patients. Future studies should focus on larger patients cohorts with non HBV etiologies, and should explore the interactions between these different etiologies to validate and extend our findings in a more general clinical setting. Fourthly, we limited our study to demographic and clinical variables. Research into molecular biological factors which influence cancer progression has begun to emerge. As a result, including these molecular factors in future studies should offer increased predictive power of our models. Finally, indicators such as C3 and PT are undergoing dynamic change, and this study only considered the patients’ baseline characteristics. Future research should include collection of dynamic data for these indicators to understand patients’ conditions and their response to treatments to a greater degree.

## Conclusion

In our research, a nomogram was developed to deliver accurate prognostic predictions for HCC patients who have undergone curative surgical resection. This predictive tool enables healthcare professionals to precisely identify individuals at elevated risk, thereby facilitating timely and appropriate therapeutic interventions.

## Data Availability

The raw data supporting the conclusions of this article will be made available by the authors, without undue reservation.
